# Characterization and Exploitation of CRISPR Loci in *Bifidobacterium longum*

**DOI:** 10.3389/fmicb.2017.01851

**Published:** 2017-09-26

**Authors:** Claudio Hidalgo-Cantabrana, Alexandra B. Crawley, Borja Sanchez, Rodolphe Barrangou

**Affiliations:** ^1^Department of Food, Bioprocessing and Nutrition Sciences, North Carolina State University, Raleigh, NC, United States; ^2^Department of Microbiology and Biochemistry of Dairy Products, Dairy Research Institute of Asturias, IPLA-CSIC, Villaviciosa, Spain

**Keywords:** CRISPR-Cas systems, genotyping, probiotics, *Bifidobacterium longum*

## Abstract

Diverse CRISPR-Cas systems provide adaptive immunity in many bacteria and most archaea, via a DNA-encoded, RNA-mediated, nucleic-acid targeting mechanism. Over time, CRISPR loci expand via iterative uptake of invasive DNA sequences into the CRISPR array during the adaptation process. These genetic vaccination cards thus provide insights into the exposure of strains to phages and plasmids in space and time, revealing the historical predatory exposure of a strain. These genetic loci thus constitute a unique basis for genotyping of strains, with potential of resolution at the strain-level. Here, we investigate the occurrence and diversity of CRISPR-Cas systems in the genomes of various *Bifidobacterium longum* strains across three sub-species. Specifically, we analyzed the genomic content of 66 genomes belonging to *B. longum* subsp. *longum, B. longum* subsp. *infantis* and *B. longum* subsp. *suis*, and identified 25 strains that carry 29 total CRISPR-Cas systems. We identify various Type I and Type II CRISPR-Cas systems that are widespread in this species, notably I-C, I-E, and II-C. Noteworthy, Type I-C systems showed extended CRISPR arrays, with extensive spacer diversity. We show how these hypervariable loci can be used to gain insights into strain origin, evolution and phylogeny, and can provide discriminatory sequences to distinguish even clonal isolates. By investigating CRISPR spacer sequences, we reveal their origin and implicate phages and prophages as drivers of CRISPR immunity expansion in this species, with redundant targeting of select prophages. Analysis of CRISPR spacer origin also revealed novel PAM sequences. Our results suggest that CRISPR-Cas immune systems are instrumental in mounting diversified viral resistance in *B. longum*, and show that these sequences are useful for typing across three subspecies.

## Introduction

Bifidobacteria are one of the first commensal microorganisms that colonize the human gut, making them the dominant intestinal bacteria in infants and one of the main inhabitants in healthy adults (Arboleya et al., 2016). The alteration in the populations of bifidobacteria present in the human microbiome has been correlated with several intestinal and immunological disorders like irritable bowel syndrome, inflammatory bowel disease (IBD), obesity, and allergy, among others (Tojo et al., 2014). The health-promoting effects of bifidobacteria consumption has shown promising results in several clinical trials for the prevention of diarrhea, reducing ulcerative colitis and IBS symptoms, and preventing necrotizing enterocolitis (Tojo et al., 2014). Among bifidobacteria, *Bifidobacterium longum* is the species most prevalence in healthy adults and widely commercialized in probiotic products. Probiotics were originally defined as “live microorganisms that, when administered in adequate amounts, confer a health benefit on the host,” (FAO/WHO., 2002; Hill et al., 2014) though a new guidance has been recently published for health claims (EFSA, 2016). Despite new regulations for health claims of probiotics, many products still misidentify the taxonomic classification of their strains based on 16S sequencing or are manufactured with low amounts of the stated microorganisms (Lewis et al., 2016; Morovic et al., 2016). In this regard, new methodologies should be applied for correct taxonomy together with internal quality control. Recently, the use of high-throughput sequencing has been suggested as a reliable methodology for correct identification (Morovic et al., 2016) as well as the use of glycolysis genes for correct taxonomy (Brandt and Barrangou, 2016).

One of the main challenges for probiotic strains is to survive the stress conditions present in the gastrointestinal tract, regarding physiological conditions (pH, bile salts, and motility) but also counteracting virus infections. The human gut constitutes a natural reservoir of phages (Stern et al., 2012), representing a huge environmental challenge for commensal and probiotic bacteria, where the need to survive constant attack has led to the need for protection against invasive DNA. One strategy that has evolved in the bacterial evolutionary arms race against foreign DNA is Clustered Regularly Interspaced Short Palindromic Repeats (CRISPR), together with CRISPR associated (*cas*) genes, that constitute the adaptive immune systems in bacteria and archaea (Barrangou et al., 2007). CRISPR-Cas systems are present in bacteria and archaea and comprise effective DNA-targeting machinery against the foreign nucleic acids (DNA and RNA) of phages and plasmids (Barrangou and Doudna, 2016). CRISPR-Cas immune systems have been widely studied and characterized during the last 10 years (Barrangou and Horvath, 2017) and, to date, two different class, six different types and numerous subtypes has been described (Makarova et al., 2011, 2015; Koonin et al., 2017; Shmakov et al., 2017). CRISPR-Cas systems are present in a wide range of microorganisms and different ecological niches, from soil to food microbes, including human commensal bacteria and also pathogens, reflecting the relevance and diversity of these immune systems.

While CRISPR technology, mainly based on CRISPR-Cas9, has been used as a genetic engineering tool with incredible popularity in eukaryotes, CRISPR has tremendous potential applications in microbiology, especially engineering food microbes, starter cultures, and probiotics (Briner and Barrangou, 2016; Hidalgo-Cantabrana et al., 2017). Moreover, the repeat-spacer arrays in CRISPR loci represent a hypervariable region that can be used for genotyping and phylogenetic studies, as well as provide insights into the immunity challenges suffered by the bacteria.

In this work, we analyzed the occurrence and diversity of CRISPR-Cas systems in *B. longum* genomes to characterize the genetic architecture of the CRISPR loci and demonstrate the potential of CRISPR-Cas systems for genotyping in this widely used probiotic species.

## Materials and methods

### CRISPR detection and identification

The 66 *B. longum* genomes (Table 1) in the GenBank database (NCBI) as of December 2016 were used to characterize the occurrence and diversity of CRISPR-Cas systems in *B. longum* strains. The CRISPR *in silico* analyses were performed as follows: the CRISPR Recognition Tool (CRT; Bland et al., 2007) implemented in Geneious 10.0.6 software (Kearse et al., 2012) was used to find the repeats sequences. Then, the Cas proteins (Cas 1, Cas 3, Cas 9) previously identified in other bifidobacteria species (Briner et al., 2015) were used as template to find the Cas proteins in the query *B. longum* strains using BLAST algorithm (Altschul et al., 1997). Afterwards, manual curation was performed to identify and annotate the correct CRISPR-Cas systems for each strain. The CRISPR subtypes designation was performed based on the signature Cas proteins and associated ones as previously reported (Makarova et al., 2011, 2015; Koonin et al., 2017).

**Table 1:** CRISPR-cas systems in *Bifidobacterium longum* strains.

***B. longum***	**Strain**	**Type-subtype**	**Repeat sequence**	**Repeat length**	**No. repeats**	***cas1***	***cas3***	***cas9***
***longum***	7	I-C	GTCGCACCCCACTGGGGTGCGTGGATTGAAAT	32	159	Y	Y	
	9	I-C	GTCGCACCCCACTGGGGTGCGTGGATTGAAAT	32	159	Y	Y	
	379	I-C	GTCGCACCCCACTGGGGTGCGTGGATTGAAAT	32	1	Y	Y	
	35624	I-C	GTCGCACCCCACTGGGGTGCGTGGATTGAAAT	32	164	Y	Y	
	105-A	II-C	CAAGCTTATCAAGAAGGGTGAATGCTAATTCCCAGC	36	34	Y		Y
	1-5B	None	None					
	1-6B	II-C	CAAGCTTATCAAGAAGGGTGAATGCTAATTCCCAGC	36	7	Y		Y
	2^nd^ locus	I-E	GGTTTATCCCCGCGTGTGCGGGGTAGAT	28	21	Y		
	17-1B	I-U	CTTGCATACGTCAAAACGTATGCACTTCATTGAGGA	36	44	Y	Y	
	2-2B	II-C	CAAGCTTATCAAGAAGGGTGAATGCTAATTCCCAGC	36	8	Y		Y
	2^nd^ locus	I-E	ACCTACCCCGCAGGCGCGGGGATAAA	26	11	Y		
	35B	II-C	CAAGCTTATCAAGAAGGGTGAATGCTAATTCCCAGC	36	12	Y		Y
	44B	II-C	CAAGCTTATCAAGAAGGGTGAATGCTAATTCCCAGC	36	18	Y		Y
	2^nd^ locus	I-E	GGTTTATCCCCGCGTGTGCGGGGTAGAT	28	25	Y		
	7-1B	II-C	CAAGCTTATCAAGAAGGGTGAATGCTAATTCCCAGC	36	34	Y		Y
	72B	None	None					
	AH1206	None	None					
	ATCC55813	None	None					
	BBMN68	I-E	GTTTGCCCCGCATGCGCGGGGATGATCCG +	29	10	Y	Y	
			GTTTGCCCCGCATGCGCGGGGATGATCCG +	29	13			
			GTTTGCCCCGCACGCGCGGGGATGATCCG	29	7			
	BG7	II-C	CAAGCTTATCAAGAAGGGTGAATGCTAATTCCCAGC	36	34	Y		Y
	BLO12	II-C	CAAGCTTATCAAGAAGGGTGAATGCTAATTCCCAGC	36	19	Y		Y
	BXY01	None	None					
	CCUG30698	None	None					
	CECT 7347	II-C	CAAGCTTATCAAGAAGGGTGAATGCTAATTCCCAGC	36	38	Y		Y
	CMCC P0001	None	None					
	CMW7750	None	None					
	D2957	None	None					
	DJO10A	II-C	CAAGCTTATCAAGAAGGGTGAATGCTAATTCCCAGC	36	43	Y		Y
	DSM 20219	None	None					
	E18	None	None					
	EK13	None	None					
	EK5	None	None					
	F8	None	None					
	GT15	None	None					
	JCM 1217	None	None					
	JDM 301	None	None					
	KACC 91563	II-C	CAAGCTTATCAAGAAGGGTGAATGCTAATTCCCAGC	36	33	Y		Y
	LMG 13197	None	None					
	LO-06	None	None					
	LO-10	None	None					
	LO-21	None	None					
	LO-C29	None	None					
	LO-K29a	None	None					
	LO-K29b	None	None					
	MC-42	I-E	GTTTGCCCCGCATGCGCGGGGATGATCCG	29	136	Y	Y	
	NCC2705	None	None					
	NCIMB8809	None	None					
	VMKB44	II-C	CAAGCTTATCAAGAAGGGTGAATGCTAATTCCCAGC	36	52	Y		Y
***infantis***	157F	None	None					
	ATCC 15697	None	None					
	BIB1401242951	None	None					
	BIB1401272845a	None	None					
	BIB1401272845b	None	None					
	BIC1206122787	None	None					
	BIC1307292462	None	None					
	BIC1401111250	None	None					
	BIC1401212621a	None	None					
	BIC1401212621b	None	None					
	BT1	I-C	GTCGCACCCCTCACGGGGTGCGTGGATTGAAAT	33	61	Y	Y	
	CCUG 52486	None	None					
	CECT 7210	None	None					
	EK3	I-E	GTTTGCCCCGCACGCGCGGGGATGATCCG	29	69	Y	Y	
	2^nd^ locus	I-C	GTCGCACCCCTCACGGGGTGCGTGGATTGAAAT	33	8	Y		
	IN-07	I-C	GTCGCACCCCTCACGGGGTGCGTGGATTGAAAT	33	61	Y	Y	
	IN-F29	I-C	GTCGCACCCCTCACGGGGTGCGTGGATTGAAAT	33	76	Y	Y	
	TPY12-1	None	None					
***suis***	AGR2137	I-E	GTTTGCCCCGCACGCGCGGGGATGATCCG	29	21	Y	Y	
		2^nd^ locus	GTCGCACCCCACTGGGGTGCGTGGATTGAAAT	32	4			
	BSM11-5	I-C	GTCGCACCCCACTGGGGTGCGTGGATTGAAAT	32	62	Y	Y	
	DSM 20211	Undet	GTCGCACCCCACTGGGGTGCGTGGATTGAAAT	32	9	N		
	LMG 21814	Undet	GTCGCACCCCACTGGGGTGCGTGGATTGAAAT	32	9	N		

### Phylogenetic analyses

Phylogenetic analyses were performed based on the amino acid sequence of Cas1, Cas2, Cas3, and Cas9 proteins, and the nucleotide sequence of the CRISPR repeats. The alignments were performed using MUSCLE algorithm (Edgar, 2004) and the trees were generated with UPGMA method (Sneath and Sokal, 1973) and 500 bootstrap replications.

### Spacers analyses

CRISPR spacers were analyzed using a custom Excel Macro tool (Horvath et al., 2008) to identify similarity between strains and their divergent evolution under DNA selective pressure. Additional studies were carried out to detect similarity between the CRISPR spacers detected in *B. longum* and prophages sequences present in bifidobacterial chromosomes, using BLASTn analyses against 190 *Bifidobacterium* genomes available at GenBank database (NCBI). Protospacers and protospacer adjacent motifs (PAM; Deveau et al., 2008; Horvath et al., 2008; Mojica et al., 2009) were defined based on these analyses, and WebLogo server was used to represent the PAM sequence based on a frequency chart were the height of each nucleotide represents the conservation of that nucleotide at each position (Crooks et al., 2004). R statistics (R Development Core Team, 2008) was used to depict the heatmaps using the “ComplexHeatmap” package (Gu et al., 2016).

## Results

### Occurrence and diversity of CRISPR in *B. longum*

**Figure 1:**
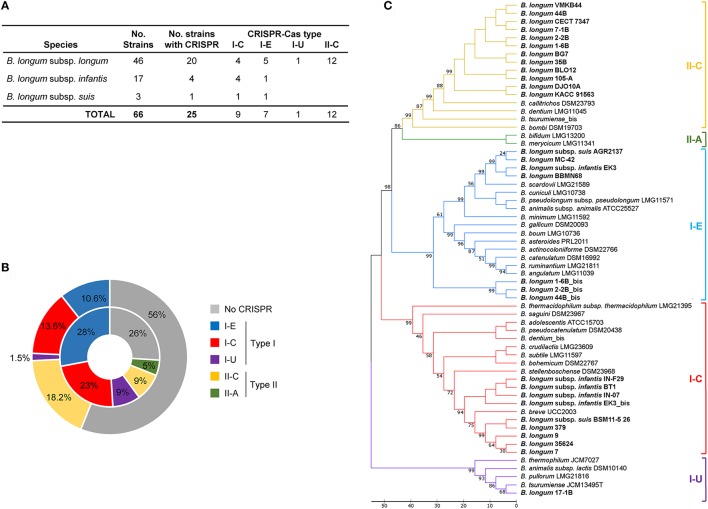
CRISPR-Cas systems in *Bifidobacterium longum*. Number of CRISPR-Cas systems detected in *B. longum* strains for each CRISPR-Cas type **(A)**. Comparison between the occurrence and diversity of CRISPR-Cas systems in *B. longum* strains (outside ring) and *Bifidobacterium* (inside ring). Percentage was calculated based on the number of positive strains for each subtype divided by total strains analyzed in each study **(B)**. Phylogenetic tree based on the amino acid sequence of Cas1 protein of *B. longum* and other bifidobacteria species, aligned with MUSCLE algorithm, and depicted with UPGMA using 500 bootstrap replicates. Bootstrap values are recorded on the nodes. The CRISPR-Cas subtypes are written on the right and groups are colored for each subtype **(C)**.

The 66 *B. longum* strains in GenBank were analyzed for the occurrence and diversity of CRISPR-Cas systems through *in silico* analyses. Initially, the presence of the universal Cas1 protein was investigated to determine the presence or absence of CRISPR-Cas systems, as Cas1 is a core protein widespread across the two main classes and six main types of CRISPR-Cas systems. Over 38% of the *B. longum* strains (25/66) harbored *cas1* genes in their genome (Table 1, Figure 1A) which is close to the 46% estimated prevalence of CRISPR in bacteria (Grissa et al., 2007). However, the occurrence of *cas1* genes in bifidobacteria species was previously described to be up to 77% (Briner et al., 2015), showing a clear difference between the genus overall and the *B. longum* species in particular. Interestingly, the strains *B. longum* 1-6B, 2-2B, 44B, and *B. longum* subsp. *infantis* EK3 encoded two *cas1* genes in a different region of the genome, representing a second CRISPR locus, a phenomenon that has been also described for other bifidobacteria strains like *B. dentium* LMG11045 (Briner et al., 2015). Overall, 29 CRISPR loci where identified in 25 strains among the three subspecies investigated, namely: *B*. *longum* subsp. *longum, B. longum* subsp. *infantis*, and *B. longum* subsp. *suis* (Figure 1A).

The CRISPR subtypes designation was performed based on the signature *cas* genes (*cas3* for Type I and *cas9* for Type II) and associated ones as previously reported for CRISPR-Cas systems classification (Makarova et al., 2011, 2015; Koonin et al., 2017). The signature *cas3* and *cas9* genes were identified in *B. longum* strains using BLAST. Overall, 12 Type II-C systems, 9 Type I-C systems, 7 Type I-E systems, and 1 Type I-U system were identified (Figure 1A). While Type I systems were detected in all three subspecies, the Type II-C selectively occurred in the *B. longum* subsp. *longum*. Moreover, CRISPR-Cas systems occurrence and diversity in *B. longum* highly differed from the distribution in *Bifidobacterium* genera (Figure 1B). Type I CRISPR-Cas systems are found in 25.7% of *B. longum* genomes whereas it they were found in 60% of bifidobacteria at the genus level (Figure 1B). In contrast, Type II systems are represented in 18.2% of *B. longum* strains, while they were only detected in 14% of the entire *Bifidobacterium* genus.

Regarding Type I systems, subtypes I-C, I-E, and I-U were identified in *B. longum*. The subtypes I-C and I-E CRISPR-Cas systems are present in the three subspecies although subtype I-C is the most common in *B. longum* subsp. *infantis*, while subtype I-E is the most prevalence in *B. longum* subsp. *longum* (Figure 1A). The CRISPR subtype I-U was only detected in *B. longum* 17-1B, and it is also present in other bifidobacteria like *B. animalis* subsp. *lactis* DSM10140, *B. pullorum* LMG21816, and *B. tsurimiense* JCM13495 (Briner et al., 2015). Interestingly, subtype I-U in bifidobacteria does not match the consensus previously described for CRISPR subtype I-U in other genera (Koonin et al., 2017), lacking *cas8*, but this genetic feature is consistent among *Bifidobacterium* genus.

Regarding Type II system, the subtype II-C is the only subtype present in *B. longum* strains, neither subtype II-A nor II-B were detected, although they are present in other bifidobacteria species (Briner et al., 2015). Noteworthy, subtype II-C was found only in the strains belonging to *B. longum* subsp. *longum*, not in subspecies *infantis* or *suis*. Indeed, subtype II-C systems is not wide-spread in bifidobacteria (Figure 1B) but it displayed high rate of occurrence in *B. longum* subsp. *longum* strains (Figure 1A).

**Figure 2:**
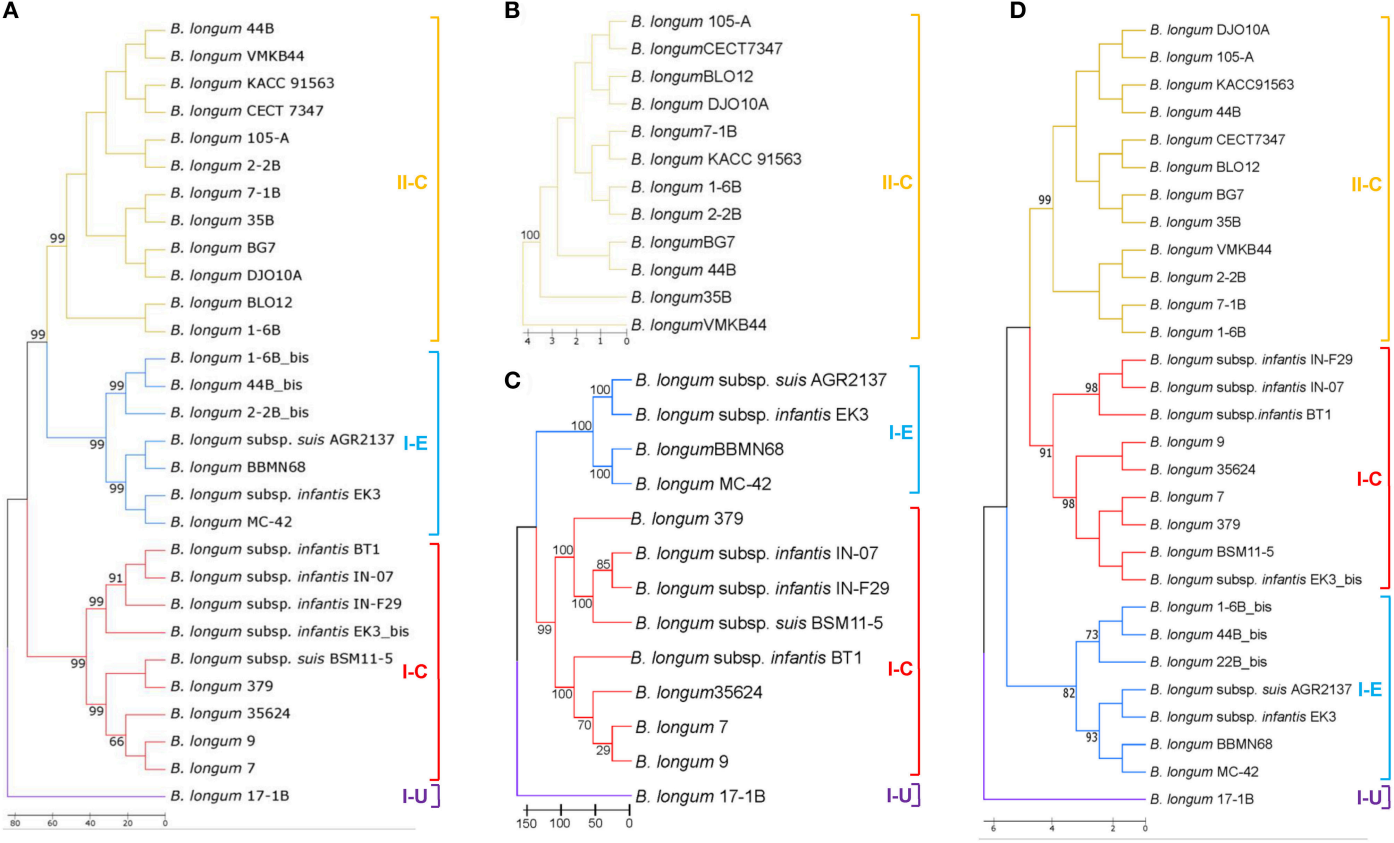
CRISPR phylogenetic analyses in *B. longum*. Phylogenetic tree based on the Cas1 protein of *B. longum* strains **(A)**, Cas9 protein **(B)**, Cas3 protein **(C)**, and the CRISPR repeats sequence **(D)**. Alignments were performed with MUSCLE algorithm and the tree was depicted with UPGMA using 500 bootstrap replicates. Bootstrap values are recorded on the nodes. The CRISPR-Cas subtypes are written on the right and groups are colored for each subtype.

The phylogenetic analyses performed with Cas1 proteins of *B. longum* and other bifidobacteria species showed the divergence of the five different CRISPR subtypes present in *Bifidobacterium* genus grouped in four major branches (Figure 1C). Type II systems (II-A, II-C) evolved from the same branch and are phylogenetically closer to subtype I-E than subtype I-C, whereas subtype I-U is more divergent. The phylogenetic analyses based on Cas1 proteins from only *B. longum* strains showed three major branches encompassing the four CRISPR subtypes detected in this species (Figure 2A), with the poorly characterized subtype I-U system segregating into its own cluster. Consistently, this clustering was also obtained for Cas2 proteins (Supplementary Figure 1), Cas9, Cas3 (Figures 2B,C) and the repeats sequence (Figure 2D), confirming the co-evolutionary trends observed in CRISPR immune systems that the components of these systems co-evolve (Makarova et al., 2011; Chylinski et al., 2014).

### CRISPR loci characterization

**Figure 3:**
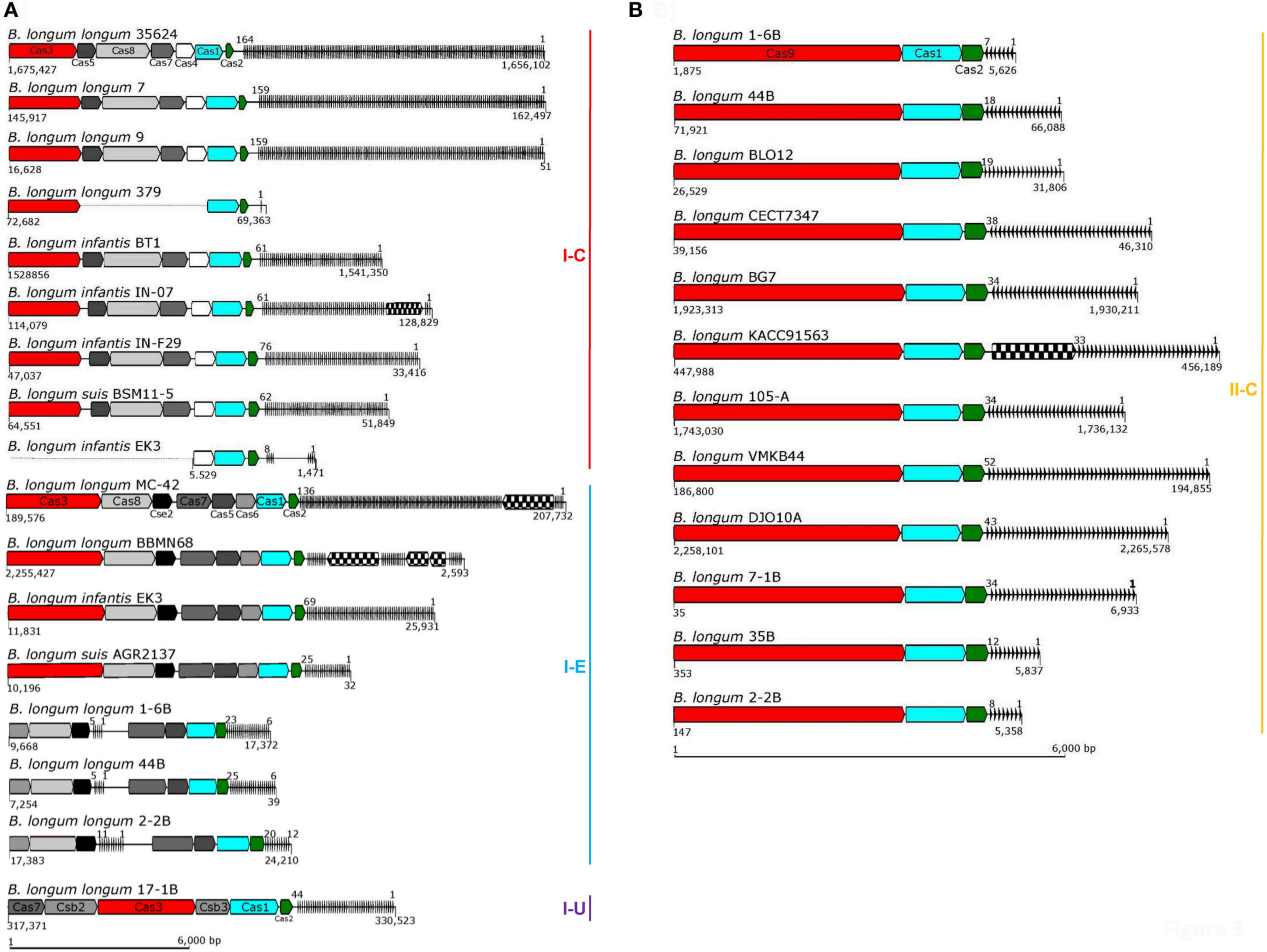
CRISPR loci in *B. longum*. The CRISPR locus of each strain was annotated and depicted with signature *cas* genes colored in red, *cas3* for Type I **(A)** and *cas9* for Type II **(B)**, and the universal *cas1* and *cas2* colored in blue and green respectively. Accessory genes are colored in a gray scale regarding their functional category, CRISPR repeats are represented as black lines on the right side of each locus (spacers are not represented) and transposase are represented with checkboard pattern fill. Numbers below CRISPR-Cas systems represent their position in the genome (or contig) and the numbers on top of the repeat-spacer array represent the number of repeats. The CRISPR loci are represented according to their size, bar scale represents 6 Kb.

The 29 CRISPR loci present in the 25 *B. longum* strains were annotated after manual curation and depicted in Figure 3. Four strains harbored two different *cas1* genes: *B*. *longum* 1-6B, 2-2B, 44B, and *B. longum* subsp. *infantis* EK3 (Table 1, Figure 3). In these four strains, the second *cas1* gene is located in a different region of the genome, together with CRISPR repeats associated *cas* genes, constituting a second putative CRISPR locus (Figure 3). However, signature *cas* genes were absent from these second loci and the type of the locus was assigned through phylogenetic clustering of the Cas1 proteins, allowing them to be subtyped by which phylogenetic clade they belonged to (Figure 2A). When multiple loci appear in the same genome, it was observed that the CRISPR subtype I-E co-occurs with the subtype II-C in the strains *B. longum* 1-6B, 2-2B, and 44B, while subtype I-C co-occurs with subtype I-E in *B. longum* subsp. *infantis* EK3. The presence of two different types of CRISPR-Cas system in the same strain has been previously described for other species like *B. dentium* LMG11045 (subtypes II-C and I-C) and *B. tsurumiense* JCM13495T (subtypes II-C and I-U; Briner et al., 2015). These incomplete CRISPR loci could be the consequence of (i) a genetic reorganization, (ii) the loss of activity of these CRISPR loci toward the acquisition of the other CRISPR loci, or (iii) incomplete assemblies indicated by the draft genomes of these strains. Moreover, the strain *B. longum* 379 displayed a truncated CRISPR locus without accessory *cas* genes, neither spacers and only one repeat (Figure 3), possibly due to genome annotation troubleshooting, thereof, this strain was exclude for the next analysis.

Regarding the size of *B. longum* CRISPR loci, subtypes I-C, and I-E varies from 12 to 18 Kb due to the genetic architecture involving several *cas* genes (multi-subunit complex Cascade) and high number of repeats (Figure 3A). Subtype II-C are the shortest loci (8 Kb), as they encompass fewer accessory *cas* genes and generally have a lower number of repeats (Figure 3B).

**Figure 4:**
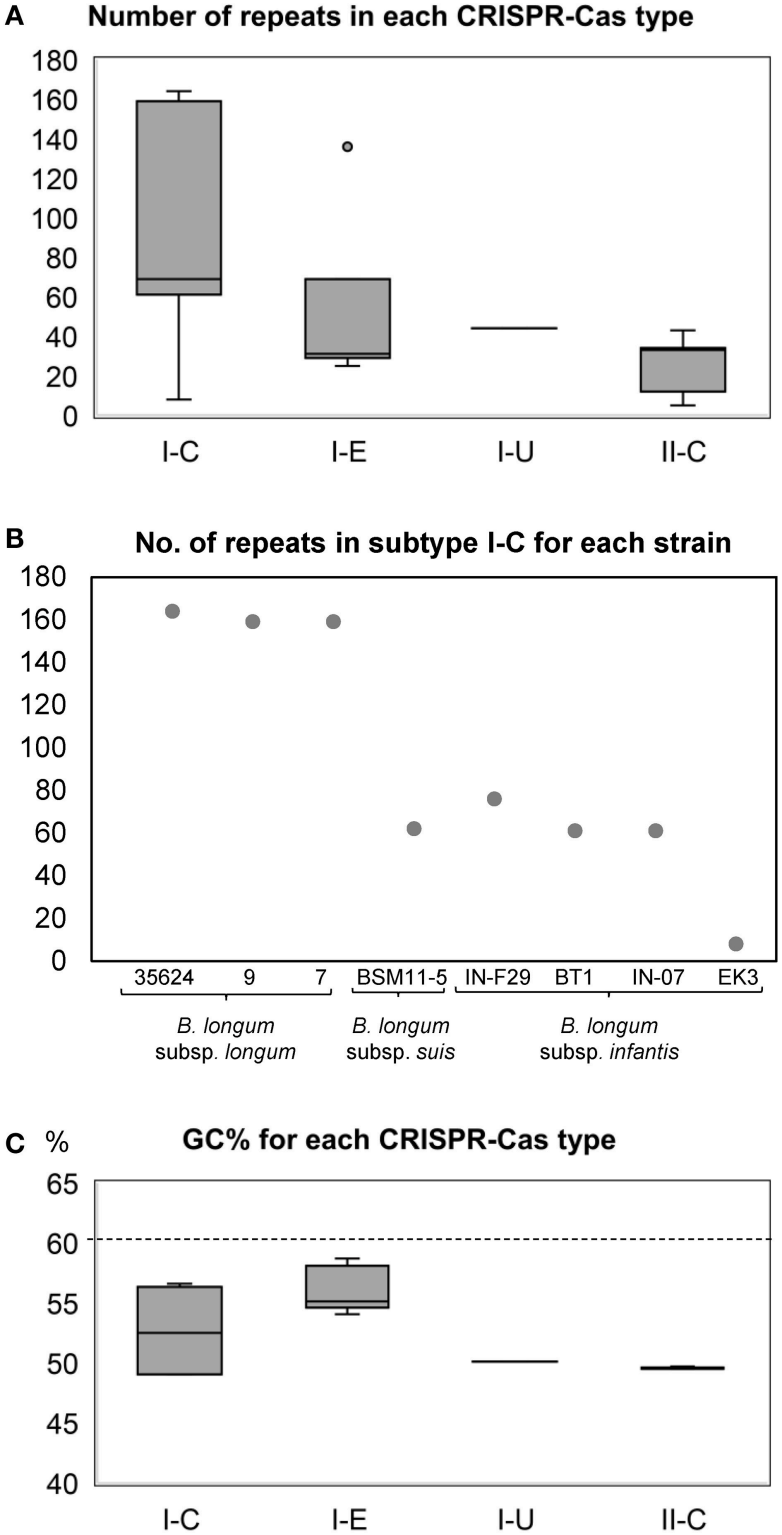
Box and Whisker representation for the number of CRISPR repeats detected in the CRISPR loci of each CRISPR subtype **(A)**. The number of CRISPR repeats in subtype I-C displayed subspecies-dependent difference between the strains of *B. longum* subsp. *longum* and *B. longum* subsp. *infantis* and *B. longum* subsp. *suis* **(B)**. CRISPR GC content and size in *B. longum*. Box and Whisker representation of the GC content of the CRISPR loci of each CRISPR subtype, dotted line represent the overall GC content of whole genome in bifidobacteria **(C)**.

Considering the repeat-spacer array size, subtype I-C varies from 61 repeats in *B. longum* subsp. *infantis* BT1 to 164 in *B. longum* 35624 (Figure 4A), with the exception of *B. longum* subsp. *infantis* EK3 displaying only 8 repeats which is likely to be related with sequencing or assembly of the locus, as the cluster appears truncated (Figure 3A). The CRISPR-Cas systems from subtype I-E presents high variability in length, ranging from 25 repeats in *B. longum* subsp. *suis* AGR2137 to 136 repeats in *B. longum* MC-42. Subtype II-C ranges from 7 repeats in the strain *B. longum* 1-6B to 52 in *B. longum* VMKB44; and the unique subtype I-U, present in *B. longum* 17-1B, contains 44 repeats (Figure 4A). Interestingly the number of repeats in subtype I-C is subspecies-dependent, with incredibly higher numbers of repeats in *B. longum* subsp. *longum* and lower in *B. longum* subsp. *infantis* and subsp. *suis* (Figure 4B).

The length of the repeats sequence is 32 nucleotides for subtype I-C, 29 nucleotides in subtype I-E, and 36 nucleotides for both subtype II-C and I-U. The repeat sequences are conserved within each CRISPR-Cas subtype in the same species, however the repeats of subtype I-C in *B. longum* subsp. *infantis* strains displayed 3 nucleotide polymorphisms (grew shadow in Table 1) compared to the consensus repeat sequence of subtype I-C in *B. longum* subsp. *longum* and *B. longum* subsp. *suis* (Table 1).

Noteworthy, transposases were found in the CRISPR loci at different locations: (i) interrupting the repeats-spacer array of subtype I-C (*B. longum* subsp. *infantis* IN-07) and subtype I-E (*B. longum* subsp. *longum* BBMN68 and MC42); (ii) between the universal *cas2* gene and the repeat-spacer array in subtype II-C (*B. longum* KACC91563). Transposases are responsible for the horizontal gene transfer that frequently occurs among prokaryotes, having an enormous impact in bacterial genomic evolution (Boto, 2010). The presence of transposases in CRISPR-Cas systems may reflect the acquisition of these genetic architectures as an evolutionary advantage to survive in a complex ecological niche like the human gut. In this regard, the GC content of the CRISPR loci was analyzed for each strain and compared to the GC content of the whole genome (Table 2). While *Bifidobacterium* spp. genomes present a high GC content, 60% average, CRISPR loci present a GC content of 50% in CRISPR subtypes I-U and II-C (all *B. longum* strains), between 54 and 58% in subtype I-E and 49 and 56% in subtype I-C (Figure 4C).

**Table 2:** *Bifidobacterium longum* strains harboring CRISPR-Cas immune systems.

***B. longum***	**Strain**	**CRISPR subtype**	**CRISPR locus GC%**	**Whole genome GC%**	**Origin**	**References**
***longum***	7	I-C	49.03	60	Commercial	Lewis et al., 2016
	9	I-C	49.03	60	Commercial	Lewis et al., 2016
	379	I-C	50.5	60.20	Human gut	Averina et al., 2012
	35624	I-C	49.03	60	Human gut	Altmann et al., 2016
	105-A	II-C	49.56	60.10	Infant feces	Kanesaki et al., 2014
	1-6B	II-C	49.6	59.6	Infant feces	Shkoporov et al., 2013
		I-E	58.11			
	17-1B	I-U	50.1	60.20	Infant feces	Chaplin et al., 2015
	2-2B	II-C	49.5	59.70	Infant feces	Shkoporov et al., 2013
		I-E	58.7			
	35B	II-C	49.5	60.10	Infant feces	Shkoporov et al., 2013
	44B	II-C	49.5	59.7	Infant feces	Shkoporov et al., 2013
		I-E	58.03			
	7-1B	II-C	49.53	59.80	Infant feces	Chaplin et al., 2015
	BBMN68	I-E	55.1	59.90	Human feces	Hao et al., 2011
	BG7	II-C	49.56	60.01	Infant feces	Kwon et al., 2015
	BLO12	II-C	49.6	60.00	Infant feces	Milani et al., 2015
	CECT 7347	II-C	49.6	60	Commercial	Chenoll et al., 2013
	DJO10A	II-C	49.55	60.11	Adult feces	Lee et al., 2008
	KACC 91563	II-C	49.7	59.81	Neonates feces	Ham et al., 2011
	MC-42	I-E	55.13	59.80	Infant feces	Tupikin et al., 2016
	VMKB44	II-C	49.6	60.30	Infant feces	Chaplin et al., 2015
***infantis***	BT1	I-C	49.3	59.4	Infant feces	Chung, 2017
	EK3	I-E	54.6	59.4	Infant feces	Chaplin et al., 2015
		I-C	55.7			
	IN-07	I-C	56.5	60.0	Infant feces	Matsuki et al., 2016
	IN-F29	I-C	56.6	59.90	Infant feces	Matsuki et al., 2016
***suis***	AGR2137	I-E	54.6	59.90	Calf feces	Kelly et al., 2016
	BSM11-5	I-C	55.8	59.90	Infant feces	Bunesova et al., 2016

### Genotyping *B. longum* strains through CRISPR spacers analyses

**Figure 5:**
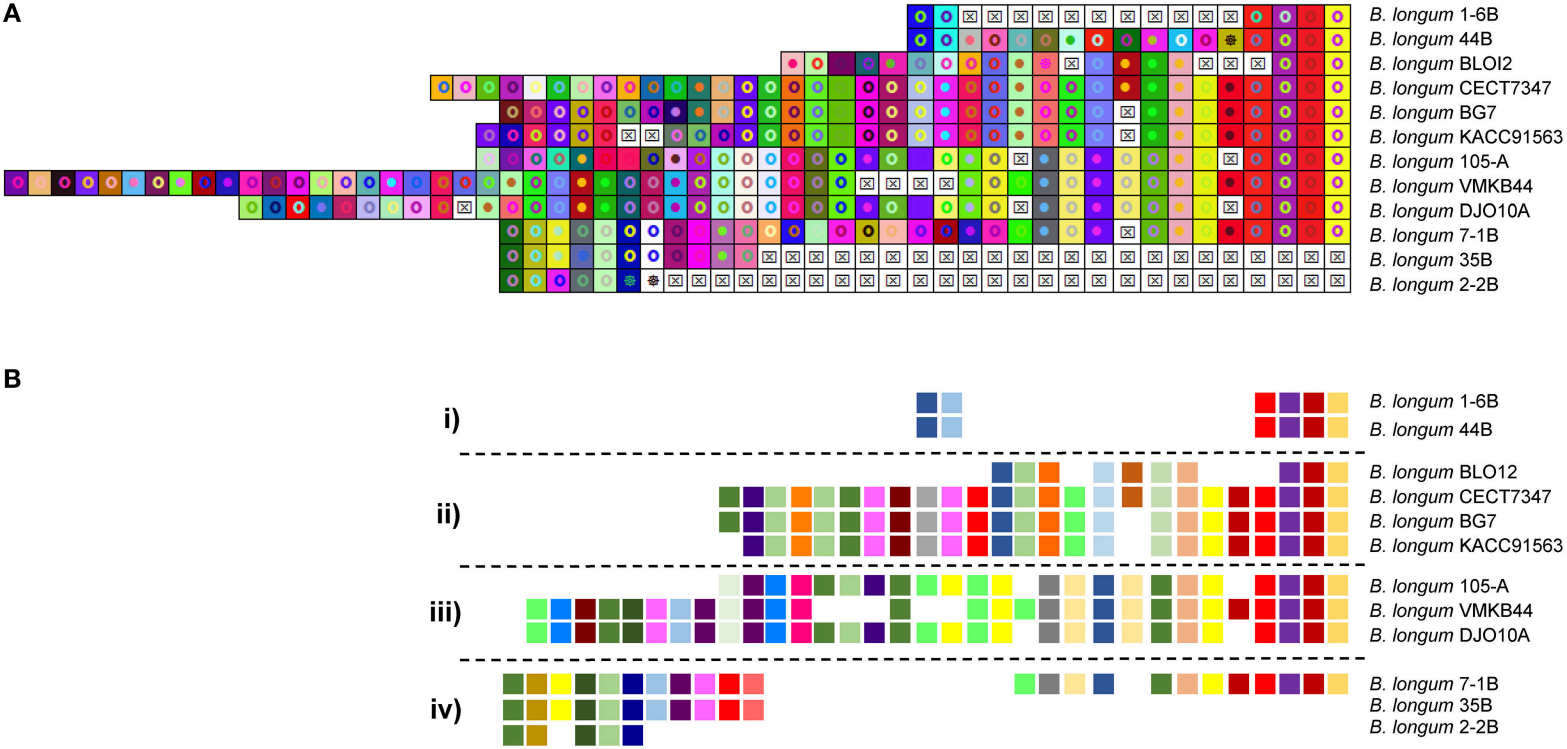
CRISPR subtype II-C spacers comparison in *B. longum*. The CRISPR spacers of CRISPR subtype II-C were represented using an Excel Macro tool. The spacers are represented by a square and each unique spacer sequence is indicated as a unique color and a geometric figure. Squares containing an “X” represent deleted or missing spacers **(A)**. The last spacer acquired is represented on the left side while the first spacer is on the right side. The spacers schematic representation showed a common origin (right side) for the strains and the evolution trend in four different clusters numbered from i to iv **(B)**.

**Figure 6:**
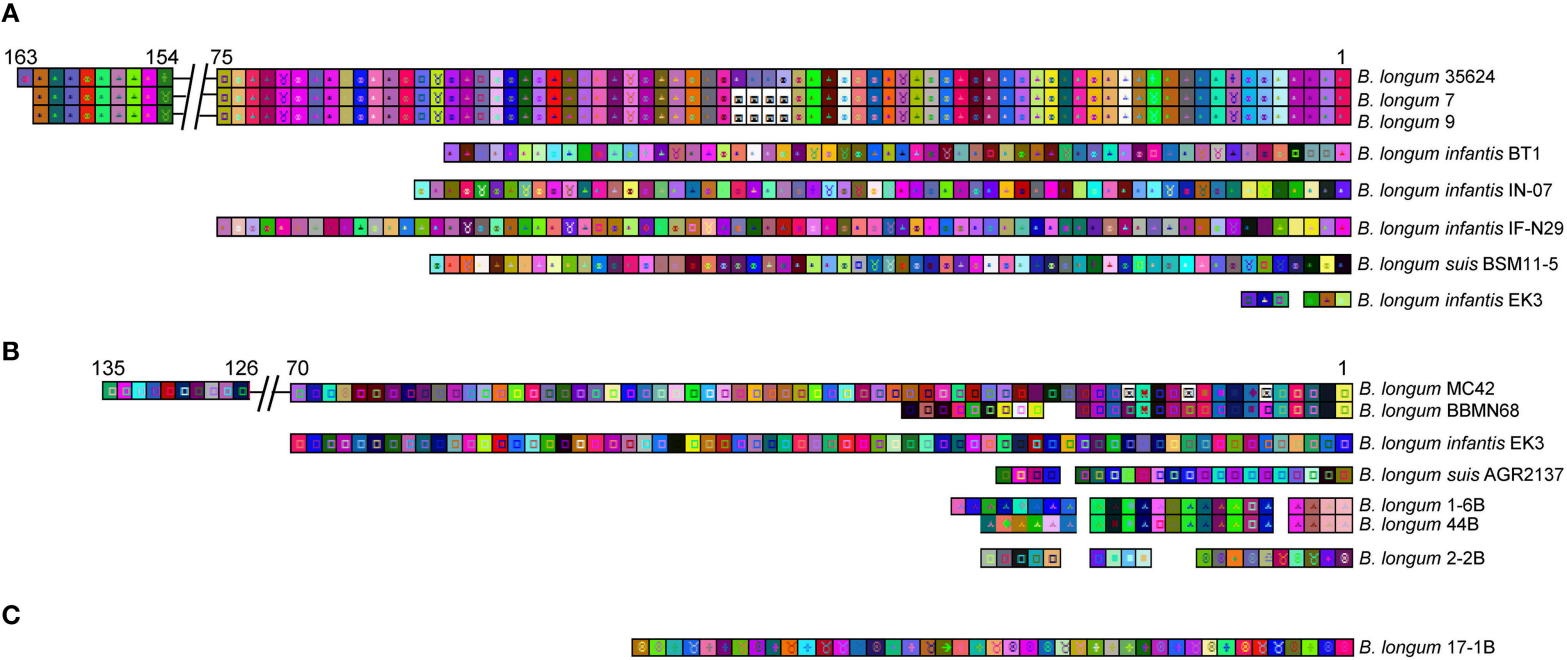
CRISPR spacers array comparison in *B. longum* for CRISPR Type I. The CRISPR spacers of CRISPR subtypes I-C **(A)**, subtype I-E **(B)**, and subtype I-U **(C)** were represented using an Excel Macro tool. The spacers are represented by a square and each unique spacer sequence is indicated as a unique color and geometric figure. Squares containing and “X” represent deleted or missing spacers. The last spacer acquired is represented on the left side while the first spacer is on the right side. Numbers on top of the spacers array indicates the first and last spacer showing the size of the array. The long arrays were reduced for a better representation and are indicated with a double line break.

The CRISPR spacers present in *B. longum* were analyzed to study the similarity and divergence among the strains based on their immunity background and their evolution under selective pressure from invasive DNA. The CRISPR spacers representation was performed based on the length and nucleotide sequence of each spacer using a “macro tool;” each unique color combination is a unique spacer sequence while the internal shape indicates the length of the spacer (Horvath et al., 2008). The CRISPR-spacer content showed diversity across and within subspecies (Figures 5, 6). For instance, analysis of the spacers from subtype II-C systems in *B. longum* subsp. *longum* revealed a common origin for the 12 strains and also reflected divergent evolution into four distinct clusters based on iterative spacer acquisition events (Figure 5B). Noteworthy, cluster i includes two closely related strains, *B longum* 44B and 1-6B, isolated from the same Russian infant (child 1) during the first year of life and 5 years later, respectively (Shkoporov et al., 2013; Chaplin et al., 2015). These two strains share ancestral and recently acquired spacers in their type II-C CRISPR systems (Figure 5) and also in Type I-E, though there are differences in recently acquired spacers in the latest timepoint (Figure 6). Moreover, cluster iv is represented by three closely related *B. longum* strains isolated from the another Russian infant (child 2) at different times over 11 years, *B. longum* 35B, 2-2B, and 7-1B (2 year old infant, 7 years and after 11 years, respectively; Shkoporov et al., 2013; Chaplin et al., 2015). These strains showed spacer conservation over the sequenced portion of the array. Furthermore, the ancestral spacers appear conserved in other strains, suggesting common ancestry, despite the individual, spatial, and temporal differences in sampling, illustrating how stable these loci are. For instance, though *B. longum* BLOI2 was isolated from an infant in Italy (Milani et al., 2015), *B. longum* KACC91563 and BG7 were isolated from Korean infants (Ham et al., 2011; Kwon et al., 2015), *B. longum* 105-A from Japanese infants, *B. longum* VMKB44 also from a Russian child from independent studies (Chaplin et al., 2015), while *B. longum* DJO10A was isolated from a healthy adult in the USA (Lee et al., 2008; Table 2).

Analyses of the spacer content in subtype I-C (Figure 6A) revealed 100% identical spacers content for the strains *B. longum* 7 and *B. longum* 9 suggesting that are the same strain, or at least share the same immunity background. Also, these two strains likely evolved from the strain *B. longum* 35624 after an internal deletion of four spacers (Figure 6A). No spacer homology was found between *B. longum* subsp. *infantis* and *B. longum* subsp. *suis* strains harboring the CRISPR subtype I-C (Figure 6A). Again, this is another example of CRISPR spacer conservation, with subtype I-E spacers (Figure 6B) shared across strains 1-6B and 44B, which were isolated form the same infant over 6 years (Chaplin et al., 2015).

### CRISPR spacers homology to prophage sequences in *Bifidobacterium*

**Figure 7:**
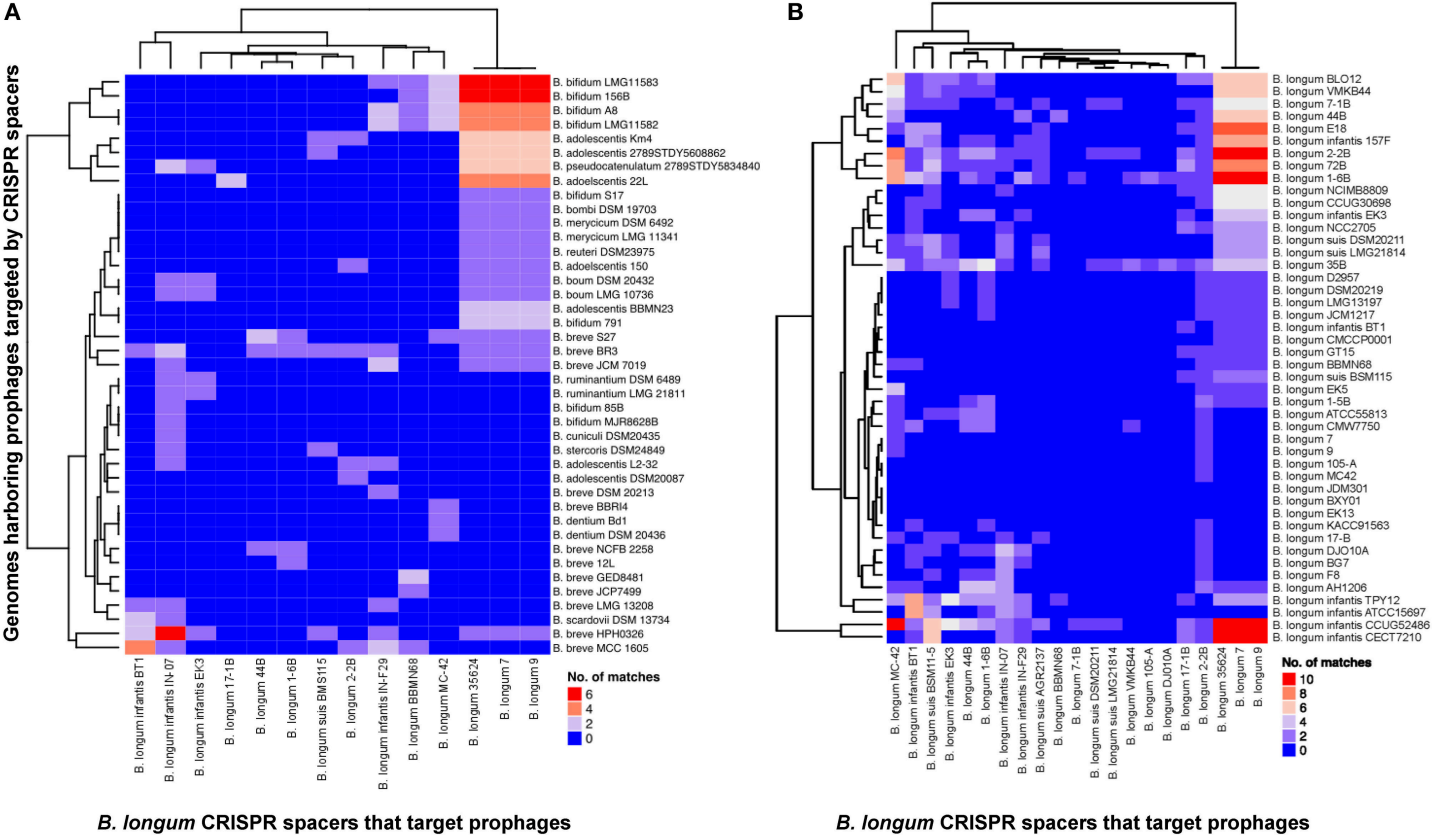
CRISPR spacers targeting prophages in *Bifidobacterium* genomes. The heatmap represents *B. longum* CRISPR spacers that matched prophages in *Bifidobacterium* genomes **(A)** and *B. longum* genomes **(B)**. The vertical axis represent the genomes that harbor prophages targeted by *B. longum* CRISPR spacers. The horizontal axis represent *B. longum* strains carrying CRISPR spacers that target prophages. The color scales represents the number of targeting events with blue squares representing the absent of matches and red squares representing the highest number of targeting.

Investigating the origin of the spacers elucidated information about the immunity record of each strain, documenting the challenges suffered and overcome against invasive DNA. The comparative analyses between the spacers present in the 29 CRISPR-Cas systems detected in *B. longum* against 190 bifidobacteria genomes revealed homology to prophages present in bifidobacterial chromosomes (Figure 7), indicating *B. longum* strains acquired immunity against prophages infecting other species, or possibly against lytic variants thereof. Interestingly, prophages in *Bifidobacterium* species were only targeted by spacers from *B. longum* CRISPR Type I systems (Figure 7A), where prophages in *B. longum* genomes where targeted by *B. longum* spacers from both Type I and Type II systems (Figure 7B).

**Figure 8:**
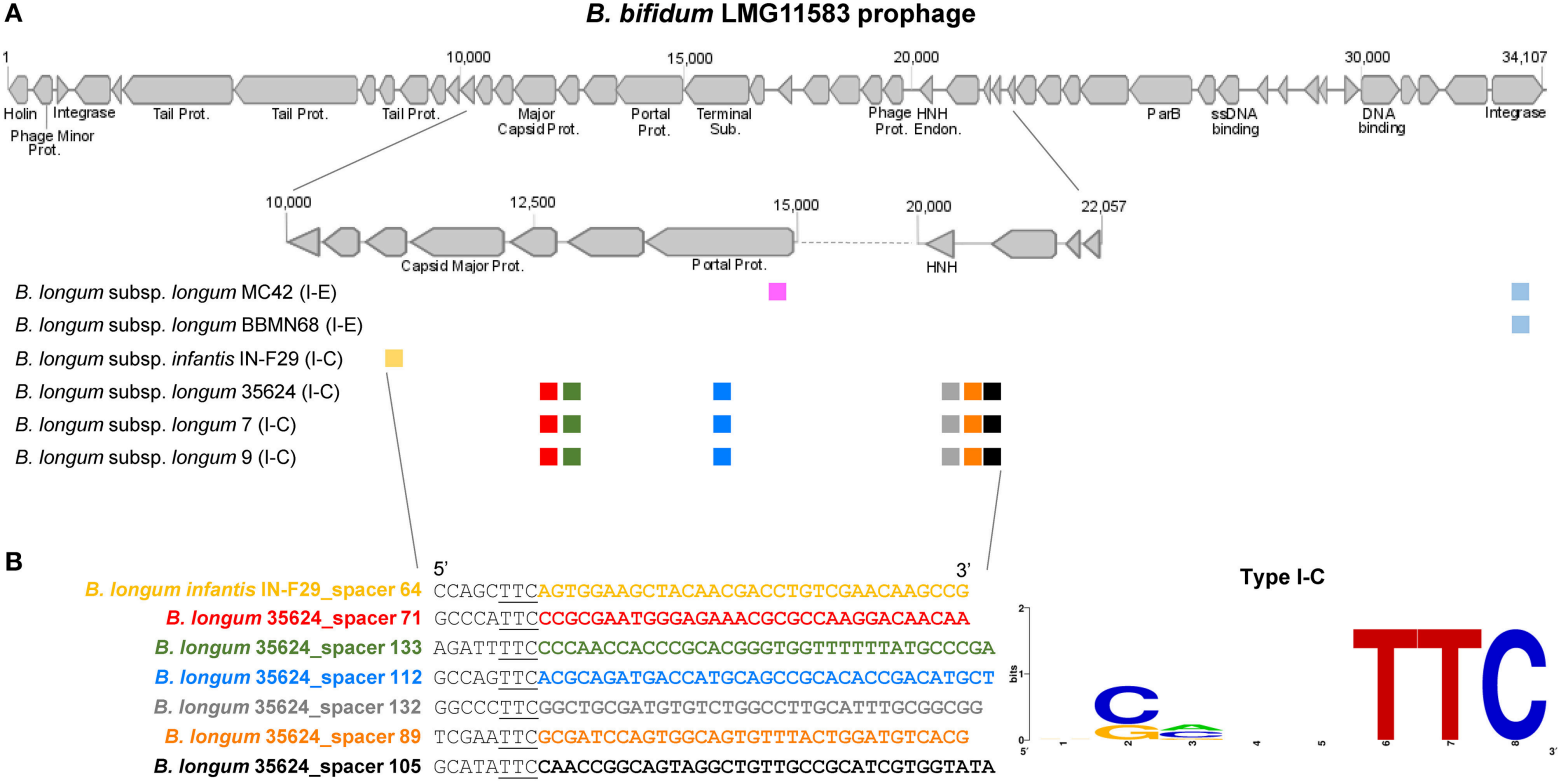
*B. bifidum* LMG11583 prophage targeted by *B. longum* spacers and PAM prediction for subtype I-C. The prophage integrated in *B. bifidum* LMG11583 chromosome is targeted by several CRISPR spacers, from *B. longum* subsp. *longum* and *B. longum* subsp. *infantis*, each unique spacer represented with a unique color, and the CRISPR-Cas subtype between brackets **(A)**. The figure on the bottom left shows the protospacer sequence of the prophage matched by each spacer (color legend) and the upstream region containing the Protospacer Adjacent Motif (PAM) underlined, whereas bottom right displayed the consensus PAM represented with the frequency plot of WebLogo server **(B)**.

From the 25 *B. longum* strains harboring CRISPR-Cas systems, 14 strains presented at least one spacer targeting a prophage in *Bifidobacteirum* genomes (Figures 7, 8). The CRISPR-Cas systems of the strains *B. longum* 35624, 7 and 9 contain the higher number of spacers targeting prophages in *Bifidobacterium* spp. genomes being *Bifidobacterium adolescentis, Bifidobacterium bifidum*, and *Bifidobacterium breve* the species most frequently targeted by CRISPR spacers of *B. longum* (Figure 7A). Moreover the strains *B. longum* 35624, 7 and 9 present also the higher number of spacers that match other *B. longum* genomes (Figure 7B) with *B. longum* subsp. *infantis* CCUG4286, CECT7210, *B. longum* subsp. *longum* 2-2B and 1-6B the strains most targeted (Figure 7B).

Regarding the diversity of the species matched by *B. longum* spacers, *B. longum* subsp. *longum* targeted prophages in up to nine different bifidobacteria species, *B. longum* subsp. *infantis* spacers targeted up to 10, whereas *B. longum* subsp. *suis* targeted only four different species (Figures 7, 8). The three *B. longum* subspecies matched prophages present in *B. adolescentis, B. breve*, and *B. longum* and differed in the other bifidobacterial species targeted (Figure 7A).

The strain *B. bifidum* LMG11583 present the most targeted prophage by *B. longum* spacers, with a total of 22 matched from nine unique spacers, from six different strains belonging to *B. longum* and *B. longum* subsp. *infantis* (Figure 8). Noteworthy, the strains *B. longum* 7, 9, 35624 present the same six spacers matching the prophage in relatively close regions of the major capsid protein and in the DNA packaging machinery components, like the portal protein and the HNH endonuclease. The portal protein plays a critical role in head assembly, genome packaging, tail attachment, and genome injection (Sun L. et al, 2015) whereas the NHN is a crucial component of the terminase packaging reaction, which is involved in packaging double-stranded DNA bacteriophage into a prohead protein (Kala et al., 2014). Thereof, the cleavage of these prophage vital components through CRISPR immune systems will prevent prophage replication and the bacteria will survive.

**Figure 9:**
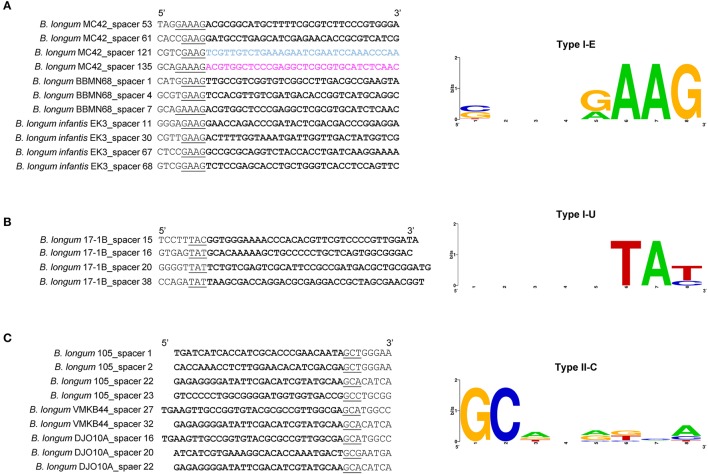
PAM prediction for *B. longum* CRISPR-Cas systems. PAM prediction for the CRISPR-Cas subtypes I-E **(A)**, I-U **(B)**, and II-C **(C)** present in *B. longum*. The left panel displayed the protospacers in bold, and the 5′-end or 3′-end (Type I and Type II respectively) containing the Protospacer Adjacent Motif (PAM) underlined. The right panel displayed the consensus PAM represented with the frequency plot of WebLogo server.

The analysis of the protospacers, the spacer sequence in the targeted DNA, together with the upstream (5′-end) and downstream (3′-end) region allowed us to define the protospacers adjacent motif (PAM; Deveau et al., 2008; Horvath et al., 2008; Mojica et al., 2009), that is absolutely necessary for DNA binding through CRISPR-Cas systems (Sternberg et al., 2014). The PAM is located immediately adjacent to the protospacer, typically at the 5′ end for Type I systems, and at the 3′ end for Type II systems, and represents a signature nucleotide sequence associated with each *cas* nuclease or effector complex. In this regard, different PAM sequences were identified for each CRISPR subtypes present in *B. longum* (Figure 9). The PAM for subtypes I-C was defined as 5′-TTC-3′, whereas the PAM for subtypes I-E was defined as 5′-NAAG-3′, and the PAM for subtype I-U was 5′-TAN-3′. Type II systems are only represented by subtype II-C in *B. longum* and the identified PAM was 5′-GCN-3′. The Cas9 identified in the twelve *B. longum* strains are 99% identical (data not shown), and therefore predicted to recognize the same PAM sequence (Figure 9). The highly conserved sequence for Cas9 in *B. longum* is also in concordance with the common origin defined for the 12 strains based on the spacers genotyping (Figure 5), and it also may reflect that these CRISPR-Cas systems are still active. The PAM identified for *B. longum* subtype II-C systems highly differs from the PAM defined for subtype II-C in *B. bombi*, 5′-NNG-3′, and from subtype II-A in *B. merycicum* 5′-NGG-3′ reflecting that Cas9 is not conserved among the different species and neither is the PAM it recognizes. Altogether, this is the first time that the CRISPR loci and the PAM has been identified for the probiotic species *B. longum*, opening new avenues for repurposing the endogenous CRISPR-Cas systems, possibly for genome editing to enhance probiotic features of these bacteria, to promote human health (Hidalgo-Cantabrana et al., 2017).

## Discussion

*B. longum* genomes showcase extensive diversity in their CRISPR-Cas systems, with variability among the three investigated subspecies (*longum, infantis*, and *suis*). Four different subtypes, belonging to Type I and Type II were detected in *B. longum* strains. Interestingly, Type I systems are present in the subspecies *B. longum* subsp. *longum, B. longum* subsp. *infantis*, and *B. longum* subsp. *suis*, although the Type II system was only detected in *B. longum* subsp. *longum* and only represented by subtype II-C. The presence of subtype II-C in *B. longum* was previously described for the strain DJO10A (Horvath et al., 2009) although it was not found in a large data set with other species of bifidobacteria (Briner et al., 2015), mainly due to the use of a unique strain as a representative of each species. Type II systems are the least common systems in nature (Makarova et al., 2015) and also in bifidobacteria (Briner et al., 2015), but it represents the highest occurrence in *B. longum* strains, although is a strain dependent characteristic and not a general feature. Noteworthy, this report showed that in bifidobacteria some of the CRISPR characteristics might be subspecies dependent, like the number of repeats and the repeat sequence, as they were different in *B. longum* subsp. *infantis* strains. A low number of repeats-spacers may reflect lower bacterial challenges against invasive DNA. The lower number of spacers detected in the CRISPR subtype I-C of *B. longum* subsp. *infantis* strains, isolated from infant feces, and high number of spacers in *B. longum* subsp. *longum* strains isolated from adult feces (Table 2), represent timing associated bacterial challenges and spacers acquisition.

The CRISPR spacer analysis of *B. longum* strains harboring the CRISPR subtype II-C allowed genotyping and evolutionary studies. The repeat-spacer array provided a hyper-variable region that could be used for genotyping purpose. The spacers displayed a common origin for all the strains suggesting they evolve from the same ancestor into four different clusters under selective pressure of invasive DNA. The spacers sequences present in the CRISPR-Cas systems of *B. longum* can be used as a genetic bar code for genotyping, showing a powerful mechanism for traceability of probiotics. The correct identification of each strains is instrumental to track select strains, to avoid misidentification, as well as to monitor and deter the potential use by competitors. This is indeed a convenient and powerful tool for the food industry to monitor and track the use and distribution of starter cultures and probiotics. Furthermore, spacer conservation in strains isolated in differences instances across individuals, location and time provides a basis for tracking genotypes with high-resolution and accuracy.

Regarding *B. longum* strains, the correct identification and taxonomy has been a problem given the genetic similarity between and within the subspecies *B. longum* subsp. *longum* and *B. longum* subsp. *infantis* (Lugli et al., 2014; Milani et al., 2014). In this regard, new genetic approaches have been proposed for high-resolution strain identification of closely related species of bifidobacteria, based on multiplex PCR primers targeting the core and variable genes (Ferrario et al., 2015) or based on terminal restriction fragment length polymorphism (Lewis et al., 2013). Recently, Lewis and co-workers showed that 15 of 16 commercial probiotic products present a bacterial composition that differ from the ingredient list, sometimes at a subspecies level (Lewis et al., 2016). Similarly, in an independent study, Morovic and co-workers showed that 42% of the commercial dietary supplements contained incorrect labeled microorganism regarding taxonomy, and 33% were below the CFU level claim (Morovic et al., 2016). Thus, alternative methodologies for genotyping and correct identification should be used in addition to traditional tools. CRISPR-Cas systems have been used for identifying: (i) industrial microbes, including: *Streptococcus thermophilus, Lactobacillus casei*, and *Lactobacillus paracasei* (Horvath et al., 2008; Broadbent et al., 2012; Smokvina et al., 2013), (ii) food pathogens: *Lactobacillus buchneri* (Briner and Barrangou, 2014) and (iii) human pathogens: *Campilobacter jejuni* (Kovanen et al., 2014), *Clostridium difficile* (Andersen et al., 2016), *Mycobacterium tuberculosis* (Sola et al., 2015; Freidlin et al., 2017), *Salmonella enterica* (Shariat et al., 2013, 2015; Bachmann et al., 2014; Almeida et al., 2017; Xie et al., 2017), *Vibrio parahaemolyticus* (Sun H. et al., 2015), *Yersinia pestis* (Barros et al., 2014; Xu et al., 2017) and *Yersinia pseudotuberculosis* (Koskela et al., 2015), among others. However, genotyping through CRISPR technologies has been seldom applied to probiotics, with few exceptions in *Lactobacillus rhamnosus* (Douillard et al., 2013) and *Lactobacillus gasseri* (Sanozky-Dawes et al., 2015). Thus, we suggest the use of CRISPR spacers as a genetic tool for genotyping *B. longum*, the most widely used probiotic species for human consumption, especially for evolutionary studies in closely related strains. However, the use of CRISPR spacers for genotyping is limited to the strains that harbor CRISPR-Cas systems in their genome.

CRISPR spacers represent the immunity record of the strain and the environmental challenges suffered with invasive DNA. In this report, we showed that *B. longum* strains displayed CRISPR spacers targeting prophages present in the genome of several bifidobacterial species. These findings are in accordance with previous reported data of prophages in the genus *Bifidobacterium* (Ventura et al., 2009; Briner et al., 2015; Lugli et al., 2016) recently named as bifidophages (Duranti et al., 2017). The high number of spacers matching prophages integrated in other bifidobacterial strains suggest that those species inhabit the same ecological niche where a co-evolution between CRISPR immune systems and prophage has occurred. The presence of CRISPR spacers in *B. longum* against certain *Bifidobacterium* spp. showed evidence of CRISPR to cause speciation, whereas the spacers matching prophages in other *B. longum* strains displayed evidence of prophage specificity. In addition, the presence of a high number of spacers in *B. longum* strains reinforce that the human gut, the main *B. longum* ecological niche, is a phage rich environment. In this sense, the human gut microbiome has been reported as a natural phage reservoir (Stern et al., 2012) where CRISPR-Cas immune systems has been detected across the human microbiome metagenomics data (Gogleva et al., 2014) and also in the oral microbiome (Wang et al., 2016). In this regard, CRISPR-Cas systems will confer an evolutionary advantage as a defense system to survive, avoiding predation by prophages and invasive DNA. Because of this, *B. longum* strains harboring CRISPR-Cas systems will be suitable probiotic candidates due to their survival capability against virus challenges based on CRISPR-Cas immune systems, ensuring their viability in the human gut and their traceability based on the spacer sequences.

Upon the characterization of CRISPR-Cas immune systems in *B. longum*, together with PAM identification, new avenues for genome engineering of next-generation probiotics are open. CRISPR technologies have led to a wide range of applications in a wide variety of organisms, although prokaryotes genome editing through CRISPR has been arguably poorly exploited to date (Hidalgo-Cantabrana et al., 2017). Genome engineering can be performed by delivering the precise, programmable and portable Cas9 nuclease in a plasmid (exogenous system) together with a single guide RNA (Jinek et al., 2012) or by repurposing the endogenous CRISPR systems of the bacteria that encode active systems, delivering self-targeting templates with a guide RNA or a CRISPR array. Briner and co-workers suggest that the CRISPR immune systems of bifidobacteria are likely active, based on preliminary transcriptomic data and complete functional CRISPR loci (Briner et al., 2015). Thus, repurposing the endogenous CRISPR-Cas systems of bifidobacteria in general, and *B. longum* in particular, provides an excellent opportunity to carry out genome editing in recalcitrant strains that are otherwise cumbersome to genetically manipulate with classical methods. Nonetheless, CRISPR technologies open new avenues to perfect probiotic bacteria and food microbes, to enhance their probiotic features, to improve their survival capability under stress conditions, or to increase their ability to modulate host immune response and impact human health.

## Conclusions

*B. longum* encode a diversity of CRISPR-Cas immune systems, belonging to four different subtypes, with large and diverse repeat-spacer arrays, indicating that these systems are likely active and protective against invasive DNA. Analysis of CRISPR spacer origin suggests adaption of this probiotic species to the human gut phage environment. Furthermore, CRISPR locus diversity shows potential for precise genotyping. The characterization of CRISPR-Cas immune systems in *B. longum* provides opportunities to develop genome editing tools using the endogenous systems for the development of next-generation probiotic bacteria.

## Author contributions

CH designed the study, performed analyses and wrote the manuscript. CH and AC performed bioinformatics analyses. RB and BS participated, coordinated, and supervised the study. All authors approved the final manuscript.

### Conflict of interest statement

RB and AC are co-inventors on several patents related to CRISPR-Cas systems and their uses. RB is a co-founder and SAB member of Intellia Therapeutics and Locus Biosciences. CH and BS are on the scientific advisory board and co-founder of Microviable Therapeutics. The other author declares that the research was conducted in the absence of any commercial or financial relationships that could be construed as a potential conflict of interest.

## Supplementary material

Supplementary Figure 1 Phylogenetic tree based on the Cas2 protein of *B. longum* strains. Alignments were performed with MUSCLE algorithm and the tree was depicted with UPGMA using 500 bootstrap replicates. Bootstrap values are recorded on the nodes. The CRISPR-Cas subtypes are written on the right and groups are colored for each subtype.
