# Supplementary figures and images for: Characterization and Exploitation of CRISPR Loci in Bifidobacterium longum
Source: Front Microbiol. 2017 Sep 26;8:1851. doi: 10.3389/fmicb.2017.01851 (PMC5626976; doi:10.3389/fmicb.2017.01851)

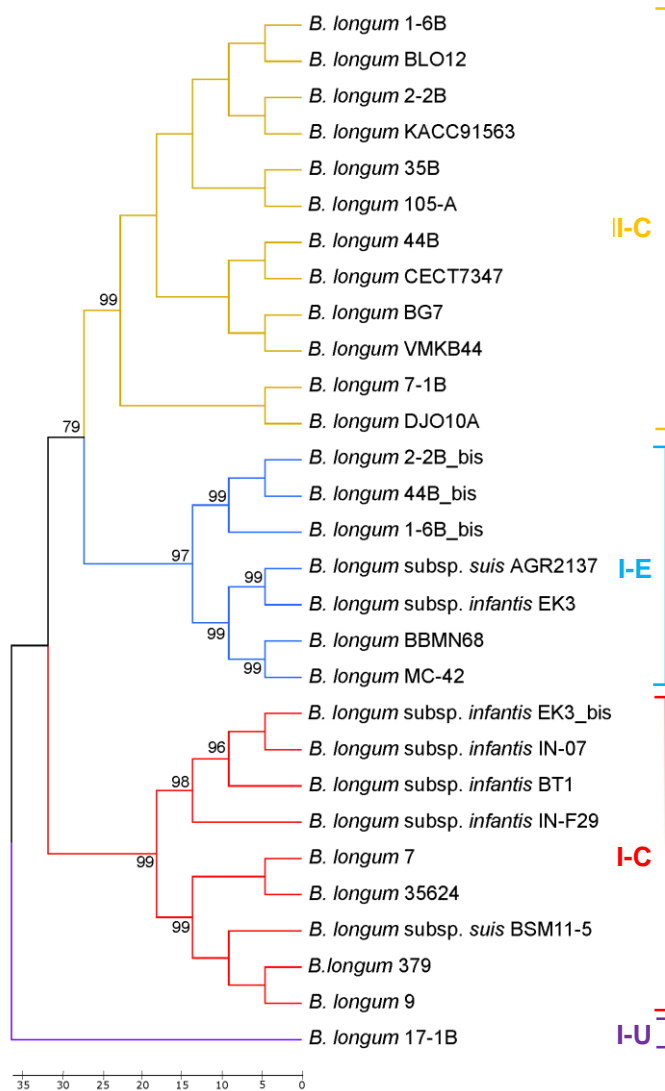

Supplementary Figure 1

Supplement: Supplementary Figure 1 — Phylogenetic tree based on the Cas2 protein of B. longum strains. Alignments were performed with MUSCLE algorithm and the tree was depicted with UPGMA using 500 bootstrap replicates. Bootstrap values are recorded on the nodes. The CRISPR-Cas subtypes are written on the right and groups are colored for each subtype. [file Image1.PDF]
